# Sustained zinc release in cooperation with CaP scaffold promoted bone regeneration via directing stem cell fate and triggering a pro-healing immune stimuli

**DOI:** 10.1186/s12951-021-00956-8

**Published:** 2021-07-12

**Authors:** Xin Huang, Donghua Huang, Ting Zhu, Xiaohua Yu, Kaicheng Xu, Hengyuan Li, Hao Qu, Zhiyuan Zhou, Kui Cheng, Wenjian Wen, Zhaoming Ye

**Affiliations:** 1 Department of Orthopedics, Musculoskeletal Tumor Center, The Second Affiliated Hospital of Zhejiang University School of Medicine, 88# Jiefang Road, Hangzhou, 310009 China; 2 Department of Thoracic Surgery, Shaoxing People’s Hospital, Shaoxing Hospital, Zhejiang University School of Medicine, No. 568 Zhongxing North Road, Yuecheng District, Shaoxing, 312000 China; 3 School of Materials Science and Engineering, Zhejiang University, Hangzhou, 310027 China

**Keywords:** Zinc-loaded CaP bioactive ceramics, Osteoimmunomodulation, Osteoinduction, Synergistic osteogenic effects, Bone regeneration

## Abstract

Metal ions have been identified as important bone metabolism regulators and widely used in the field of bone tissue engineering, however their exact role during bone regeneration remains unclear. Herein, the aim of study was to comprehensively explore the interactions between osteoinductive and osteo-immunomodulatory properties of these metal ions. In particular, the osteoinductive role of zinc ions (Zn^2+^), as well as its interactions with local immune microenvironment during bone healing process, was investigated in this study using a sustained Zn^2+^ delivery system incorporating Zn^2+^ into β-tricalcium phosphate/poly(L-lactic acid) (TCP/PLLA) scaffolds. The presence of Zn^2+^ largely enhanced osteogenic differentiation of periosteum-derived progenitor cells (PDPCs), which was coincident with increased transition from M1 to M2 macrophages (M$\varphi$s). We further confirmed that induction of M2 polarization by Zn^2+^ was realized via PI3K/Akt/mTOR pathway, whereas marker molecules on this pathway were strictly regulated by the addition of Zn^2+^. Synergically, this favorable immunomodulatory effect of Zn^2+^ further improved the osteogenic differentiation of PDPCs induced by Zn^2+^ in vitro. Consistently, the spontaneous osteogenesis and pro-healing osteoimmunomodulation of the scaffolds were thoroughly identified in vivo using a rat air pouch model and a calvarial critical-size defect model. Taken together, Zn^2+^-releasing bioactive ceramics could be ideal scaffolds in bone tissue engineering due to their reciprocal interactions between osteoinductive and immunomodulatory characteristics. Clarification of this synergic role of Zn^2+^ during osteogenesis could pave the way to develop more sophisticated metal-ion based orthopedic therapeutic strategies.

**Supplementary Information:**

The online version contains supplementary material available at 10.1186/s12951-021-00956-8.

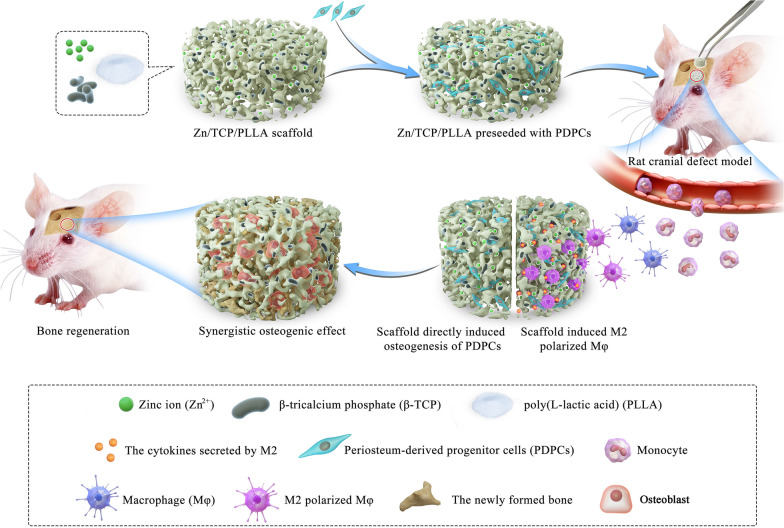

## Introduction

To date, the recovery from large segmental bone defects remains a great challenge in the clinical field of orthopedics [1]. When the defect exceeds a critical size, the self-healing capabilities of the body are insufficient to completely repair it [2]. Bone grafts are the most popular approaches for the clinical treatment of bone defects in such cases [3]. However, due to the insufficient supply of and various limitations related to autologous and allogeneic grafts and xenografts (such as immune rejection, donor site morbidity, communicative diseases and risk for infection) [4], osteogenic biomaterials are urgently needed as bone graft substitutes to satisfy persisting healthcare needs [5, 6].

Zinc (Zn), one of the most important trace elements in bone tissues [7], has been widely reported to have a stimulatory effect on the osteogenic differentiation of stem cells [8–10]. β-Tricalcium phosphate (β-TCP), a kind of bioceramic, has been commonly used as a suitable carrier to deliver ions/biomolecules/drugs [11]. Thus, Zn-loaded β-TCP (Zn/TCP) might be a potential scaffold with the property of sustained releasing Zn ion (Zn^2+^) for bone repair. However, TCP’s inherent brittleness and low mechanical properties largely hinder its direct application in the load-bearing area of bone [12]. It is also quite difficult to maintain and shape TCP scaffolds in defect sites [13]. To address this issue, poly(L-lactic acid) (PLLA) was selected as the substrate material for Zn/TCP incorporation. PLLA, a biodegradable polymer, has been approved by the United States Food and Drug Administration for application in tissue engineering due to its excellent biocompatibility and biodegradability [14]. Recent studies found that PLLA is biocompatible with cell attachment, proliferation and differentiation, and bone regeneration [15–17]. Recently, Zinc loaded porous materials has been widely applied for several areas such as biomedicine, biotechnology, and analytical chemistry [18, 19]. In this study, the porous and degradable properties of PLLA are perfectly suitable for exposing wrapped Zn/β‐TCP particles and for the sustained release of Zn^2+^ into the surrounding bodily fluid. In the current study, Zn/TCP was incorporated into PLLA to form Zn/TCP/PLLA scaffolds for bone defect repair. We believe that Zn/TCP/PLLA scaffolds could promote bone healing by directly inducing the osteogenic differentiation of stem cells.

With the development of an understanding of the reactions between biomaterials and host tissues, the regulatory effect of the immune system on biomaterial-induced osteogenesis has gradually become a focus of study [20, 21]. To date, most studies have mainly identified the direct osteoinductive effect of Zn^2+^ [8, 22] or β-TCP [23, 24] on host/exogenous stem cells. Whether and how the Zn/TCP/PLLA scaffold regulates the local osteoimmune microenvironment around the bone defect area remains unknown. Macrophages (M$\varphi$s), one of the major multifunctional effector cells of the immune system, have been reported to play an indispensable role in the regulation of bone healing [25]. The plasticity and heterogeneity of M$\varphi$s make them a prime target for immune system modulation to promote bone repair and regeneration [26]. Specifically, under the modulation of the microenvironment created by the implanted scaffolds, M$\varphi$s undergo polarization to either the anti-inflammatory (M2 subtype) or pro-inflammatory (M1 subtype) phenotype and subsequently release a wide series of bioactive molecules, leading to active regeneration of bone or induction of persistent inflammation, respectively [26, 27]. Recently, several studies proposed that Zn-decorated membranes or Ti/TiO_2_ implants could intensively induce the polarization of M$\varphi$s toward the anti-inflammatory phenotype (M2) [28–30]. Similarly, Zn^2+^ deficiency aggravated inflammation, reduced the numbers of M2-polarized M$\varphi$s and increased the numbers of M1-polarized M$\varphi$s [31, 32]. In addition, β-TCP was also reported to possess the ability to shift M$\varphi$ polarization toward the M2 subtype [33–35]. M2-polarized M$\varphi$s guide MSC differentiation along the osteogenic pathway and subsequently increase bone mineralization [36, 37]. Hence, it is probable that the Zn/TCP/PLLA scaffold could facilitate a favorable osteoimmunomodulatory response that directionally induces the M2 polarization of M$\varphi$s, resulting in enhanced MSC-associated osteogenesis. Moreover, this favorable osteoimmunomodulation effect coupled with the spontaneous osteogenesis of Zn/TCP/PLLA scaffolds might further enhance bone regeneration.

In summary, this study aimed to fabricate Zn^2+^-loaded bioactive ceramics seeded with periosteum-derived progenitor cells (PDPCs) for repair of critical-sized bone defects and mainly focused on whether there are synergetic osteogenic effects of spontaneous osteogenesis and favorable osteoimmunomodulation of this scaffolds on bone regeneration. In the current study, PDPCs and M$\varphi$s were independently cultured on the surface of Zn^2+^ loaded CaP scaffolds to determine the osteoinductive and osteo-immunomodulatory properties of the sustained released Zn^2+^ in vitro, respectively. Next, PDPCs were seeded on scaffolds and cultured with the corresponding scaffold-induced M$\varphi$-conditioned medium to explore the synergistic effects of the Zn^2+^ loaded CaP scaffolds and scaffold-induced pro-healing immune stimuli of M$\varphi$s on the bone regeneration in vitro. Moreover, the immunomodulatory and osteogenic effects of Zn^2+^ loaded CaP scaffolds in vivo were further identified using a rat air pouch model and a calvarial critical-size defect model, respectively.

**Fig. 1:**
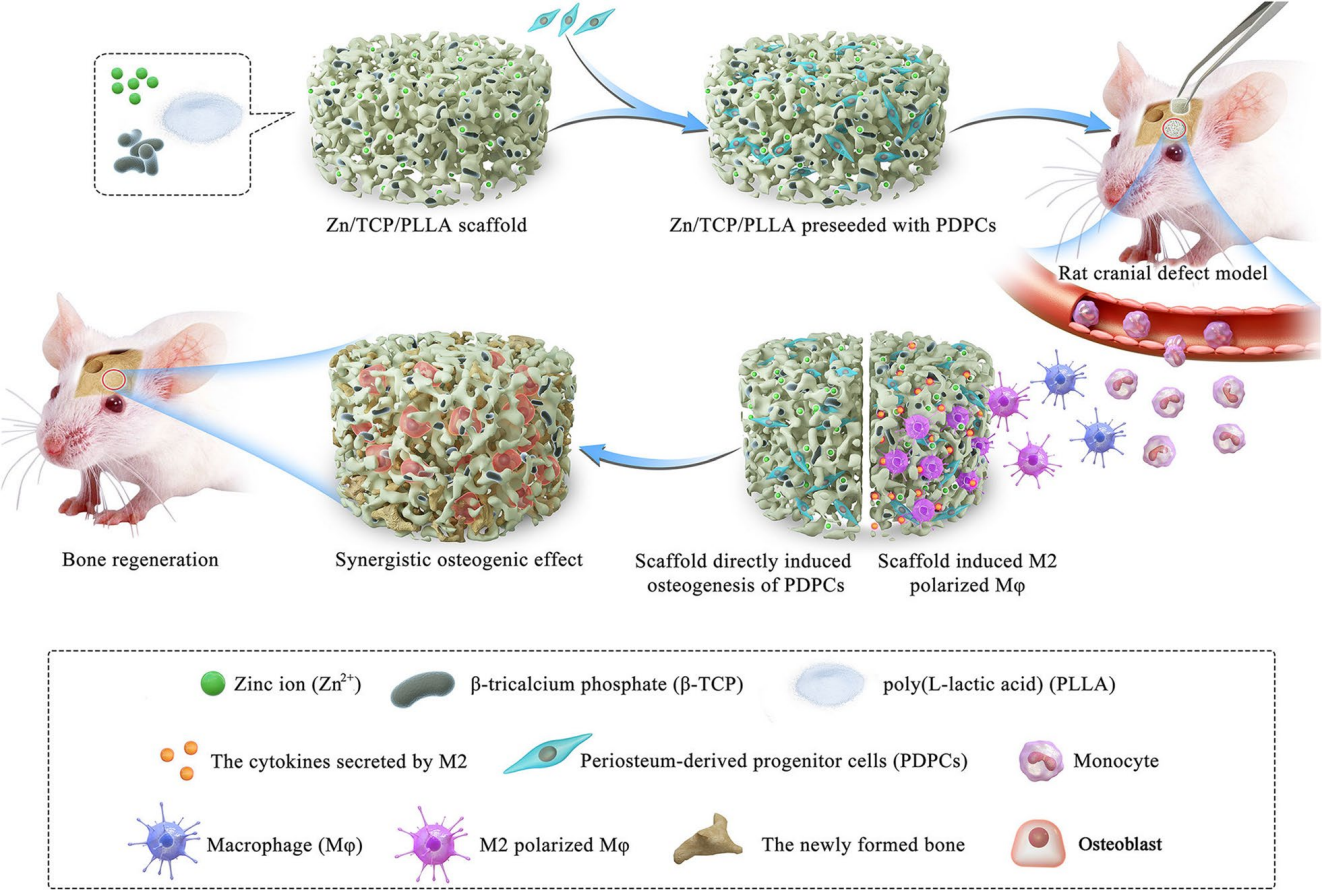
Schematic illustration of how Zn-loaded bioactive ceramics contributes to endogenous bone regeneration: Zn-loaded bioactive ceramics directly induces PDPC osteogenic differentiation and promotes M2 polarization, which subsequently further enhance PDPC-mediated bone formation

We hypothesized that the Zn-loaded bioactive ceramics not only directly induced bone regeneration by promoting PDPC osteogenic differentiation but also generated a local immune microenvironment favorable to promoting PDPC osteogenesis by specifically inducing M2 polarization of M$\varphi$s (Fig. 1). Our results demonstrate the unique scaffold implantation strategies of combining spontaneous osteogenesis with favorable osteoimmunomodulation to potentially improve bone regeneration capacity.

## Materials and methods

### Preparation of Zn/TCP/PLLA composites and characterizations

The Zn/TCP/PLLA were fabricated as described previously [38, 39]. Briefly, Zn-containing TCP particles were produced by wet precipitation method using H_3_PO_4_ (AR) and Ca(OH)_2_ (AR) at a Ca/P molar ratio of 1.5 and dissolved in deionized water and mixing 0.5 M Zn(NO_3_)_2_ · 4H_2_O (AR) at molar percentages of 0%, 5% and 10% (Zn/(Zn + Ca)), respectively. PLLA (M_w_ = 200,000; Chengdu Institute of Organic Chemistry, Chinese Academy of Sciences) was dissolved in 5% Pluronic F127, and TCP particles containing 0%, 5% and 10% Zn were added at a percentage of 50 wt%. After 1 h of strong stirring at 80 °C, the mixture was frozen for 8 h at − 5 °C and lyophilized in a freeze-dryer for 72 h. After sterilization by ethylene oxide, the porous scaffolds were stored at room temperature. TCP/PLLA scaffolds prepared with 0%, 5% and 10% Zn were denoted “TCP”, “5%Zn” and “10%Zn”, respectively, in the following manuscript and figures. We also prepared PLLA scaffolds without TCP particles as a control group (denoted “PLLA”). The microstructure and morphology of Zn-containing TCP particles were detected by transmission electron microscopy (TEM, JEOL 1200; JEOL, Tokyo, Japan). The porosities of scaffolds were determined by ethanol replacement method as the previous study reported [40].

### Zn^2+^ release test

The layer specimens (10 mm × 10 mm × 1 mm) were immersed in 10 ml tris-buffer solution (0.05 M, pH = 7.3, at 37 ℃) in sealed polyethylene tubes. After incubation for 0.5, 1, 3, 7, 10 and 14 days, the concentration of Zn^2+^ in the immersed solution was analyzed by atomic absorption spectrophotometry (HITACHI 180–50; Hitachi, Tokyo, Japan).

### Scanning electron microscopy (SEM)

The surface morphologies and distributions of PDPCs seeded on the scaffolds were observed using SEM (Hitachi Model TM-1000; Hitachi). Cells were seeded at a density of 1 × 10^4^ cells per well and harvested at 1 day and 3 days. Then, specimens were fixed in 2.5% glutaraldehyde solution and washed 3 times by phosphate-buffered saline (PBS). Next, the specimens were immersed in OsO_4_ in PBS for 1 h, washed 3 times by PBS (10 min each) and dehydrated with a graded series of ethanol (30–100% v/v) for 10 min each. After immersion in pure isoamyl acetate overnight, the specimens were dried by critical point drying method and mounted on aluminum stubs, coated with gold powder, and observed under SEM at an accelerating voltage of 1.0 kV.

### Isolation of PDPCs

PDPCs were harvested from the femurs of Sprague–Dawley (SD) rats (male, 4 weeks old). First, aseptically remove the muscle and connective tissue from the femurs. Next, the femurs were dissected, cut into small pieces (1–2 × 1–2 mm), and embedded in 5% low melting point agarose (Takara, Shiga, Japan) to prevent them from digestion. PDPCs were harvested by 0.2% collagenase digestion for 1 h at 37 °C. Single-cell suspensions were plated onto a 60-mm culture dish in Dulbecco’s Modified Eagle’s Medium–low glucose (DMEM, Gibco) with 10% (vol./vol.) fetal bovine serum (FBS; Gibco) and 1% (vol./vol.) penicillin/streptomycin (Gibco) at 37 °C with 5% CO_2_. The culture medium was changed every 3 days. The cells were used between passages 3 and 5.

### FACS analysis

The cells (5 × 10^5^) were washed 3 times by PBS and then incubated with 10 μL of fluorescein isothiocyanate (FITC)- or R-phycoerythrin (PE)-conjugated mouse anti-rat monoclonal antibodies against the following surface antigens: CD29, CD31, CD44, CD45, CD79, and CD90 (BioLegend, San Diego, USA). Next, the samples were detected by a FACSCanto flow cytometer (BD Biosciences, San Jose, USA). The data were analyzed by FlowJo software.

### Trilineage differentiation

The differentiation potential of the PDPCs at the third passage to the osteogenic, adipogenic and chondrogenic lineages was explored. In brief, the osteogenic differentiation of PDPCs was induced by DMEM with 10% FBS, 50 μM ascorbic acid, 1% L-glutamine, 10 mM β-glycerol phosphate, 1% NEAA, and 0.1 μM dexamethasone. Alizarin red S was applied to detect the mineral deposition. Chondrogenic differentiation was induced in PDPCs micromass culture with chondrogenic differentiation medium, consisting of DMEM with 1% FBS, 0.1 μM dexamethasone, 1% L-glutamine, 1% NEAA and supplemented with 1% ITS, 40 μg/mL L-proline, 50 μM ascorbic acid, 10 ng/mL transforming growth factor 3, and 1% sodium pyruvate. Safranin O was applied to evaluate positive induction. Adipogenic differentiation was induced by DMEM with 10% FBS, 0.5 μM dexamethasone, 1% L-glutamine, 1% NEAA, 10 μg/mL insulin, 0.5 mM isobutylxanthine, and 100 μM indomethacin. Oil red was applied to detect lipid accumulation.

### Colony formation assay

PDPCs were seeded at 200 cells per 6-cm dish to form colonies. After 2 weeks of culture, the cells were washed twice with PBS, stained with 0.75% g/mL crystal violet solution for 30 min, and then washed 5 times with distilled water. Finally, photos of dried cells were captured by a bright-field microscope (Leica, Wetzlar, Germany).

### Preparation of M$\varphi$conditioned medium

RAW 264.7 cells were purchased from the Cell Bank of the Chinese Academy of Sciences (Shanghai, China). The cells were seeded into the scaffolds with complete culture medium (DMEM, Gibco) supplemented with 1% (v/v) penicillin/streptomycin (Invitrogen) and 10% FBS (Gibco)) at 37 ℃ in a humid atmosphere with 5% CO_2_. After 3 days of culture, the M$\varphi$ conditioned medium was collected and mixed with complete medium at a 1:1 ratio and stored at − 80 ℃ for subsequent use.

### Cell proliferation assay

The proliferation of PDPCs and RAW 264.7 cells was measured with the Cell Counting Kit-8 (CCK-8, Dojindo, Japan). The PDPCs were cultured on the scaffold in 24-well plates at a density of 1 × 10^4^ cells per well for 2, 4 and 6 days and were then incubated in 10% CCK-8 solution at 37 °C for 2 h in the dark. The RAW 264.7 cells were cultured on the scaffold in 24-well plates at a density of 1 × 10^4^ cells per well for 1 and 3 days and were also incubated in 10% CCK-8 solution under the same conditions. The absorbance of the culture medium at 450 nm was detected using a microplate reader. Cell viability were correlated with absorbance value. Each experiment was repeated in triplicate.

### Live/dead fluorescence staining

On days 6 and 3 after seeding PDPCs and RAW 264.7 cells, respectively, the cells were rinsed with PBS buffer solution, and the viability was determined by the live/dead viability/cytotoxicity kit (Beyotime, Shanghai, China) according to the manufacturer’s protocol. Briefly, the cells were incubated for 20 min at room temperature in a mixture of two probes: calcein AM and PI. The live and dead cells were then photographed under a laser scanning confocal microscope (Olympus FV1000).

### Quantitative real-time reverse transcriptase polymerase chain reaction (qRT-PCR)

The mRNA levels of bone morphogenetic protein-2 (BMP-2), Smad1, alkaline phosphatase (ALP), and osteocalcin (OCN) in PDPCs cultured on scaffolds, cultured on normal plates in conditioned media from RAW264.7 cells, and cultured on scaffolds in conditioned media from RAW264.7 cells were evaluated by qRT-PCR. Total RNA was extracted using TRIzol reagent (Takara) according to the manufacturer's instructions. Then, cDNA was synthesized from 1 μg of RNA by a SYBR Premix Ex Taq kit (Takara). Real-time PCR was conducted using SYBR GREEN Master Mix (Takara) on an ABI StepOnePlus System (Applied Biosystems, Warrington, UK). The primer sequences were listed in Table 1. All the experiments were performed in triplicate independently. The relative gene expression levels were normalized to the value of GAPDH by the delta-delta cycle threshold (ΔΔCT) method.

**Table 1:** Summary of primers used in quantitative reverse transcription polymerase chain reaction

Gene	5–3'	Primers
Rat-BMP-2	Forward	GATCTGTACCGCAGCCACTCA
	Reverse	AAGCTTCCTGTATCTGTTCCCG
Rat-Smad1	Forward	GGATGAGCTTCGTGAAGGGTTGG
	Reverse	GCAAGAGACGGAAGCCACAGG
Rat-ALP	Forward	CGGCCATCCTATATGGTAACGG
	Reverse	CAGGAGGCATACGCCATCACA
Rat-OCN	Forward	GACCCTCTCTCTGCTCACTCT
	Reverse	GACCTTACTGCCCTCCTGCTTG
Rat-GAPDH	Forward	GCAAGTTCAACGGCACAG
	Reverse	CGCCAGTAGACTCCACGAC

*BMP-2* Bone Morphogenetic protein-2, *ALP* alkaline phosphatase, *OCN* osteocalcin, *GAPDH* glyceraldehyde 3‐phosphate dehydrogenase

### Immunofluorescence

Scaffolds were inoculated with RAW264.7 cells, and after 3 days, the scaffolds were washed twice with PBS buffer solution and fixed in 4% paraformaldehyde for 20 min at room temperature. Then, the membranes were disrupted with 0.4% Triton X-100 (PBS configuration) for 15 min. Next, the cells were blocked for 1 h using PBS buffer solution containing 1% bovine serum albumin and 2% goat serum. The cells were subsequently incubated with CD206 antibody (1:1000, Abcam, ab64693, UK)/iNOS antibody (1:500, Abcam, ab210823, UK) in a humidified chamber overnight at 4 °C. After that, the cells were incubated with the respective secondary antibodies for 2 h and counterstained with 5 μg/ml 4′,6‐diamidino‐2‐phenylindole (DAPI) in a dark room for 15 min. The stained samples were photographed by laser scanning confocal microscopy (Olympus FV1000).

### Enzyme-linked immunosorbent assay (ELISA)

Scaffolds were inoculated with RAW264.7 cells, and after 3 days, the culture medium was collected. The levels of the inflammatory cytokines IL-1β, TNF-α, and IL-10 in the cell culture medium were measured by commercially available ELISA kits (Neobioscience Technology Co., Ltd., Hong Kong, China) according to the manufacturer’s instructions. The absorbance was detected at 450 nm on a full wavelength microplate reader (Bio-Rad).

### Alizarin Red staining

After incubation of PDPCs in M$\varphi$ conditioned medium for 3 weeks, alizarin red staining was applied to evaluate osteogenic potential. The cells were rinsed with PBS and fixed in 2% paraformaldehyde for 15 min. Next, 40 mM Alizarin Red S (pH 4.1 to 4.3, Sigma-Aldrich) was added to stain the cells for 15 min at room temperature. The cells were rinsed in triplicate with deionized water and photographed. After that, 10% cetylpyridinium chloride destain solution was added to each well and incubated at room temperature for one hour. At last, samples were transferred to a 96-well plate, and the absorbance at 560 nm was measured in triplicate by a full wavelength microplate reader (Bio-Rad).

### Western blot

M$\varphi$s were incorporated onto TCP/PLLA and 5%Zn/TCP/PLLA and treated with or without the PI3K inhibitor LY-294002 (40 μM). After 3 days, M$\varphi$s were collected and lysed in lysis buffer (Nanjing Jiancheng Bioengineering Institute) on ice using a western and IP cell lysis kit. Next, the protein extracts were collected by centrifugation at 15 000*g* for 20 min at 4 °C. The protein concentrations of the cell lysates were tested using the Bio-Rad protein assay (Beyotime) and read on a microplate reader at 595 nm. The prepared protein solutions from each sample were loaded and run on a 10% sodium-dodecyl sulfate polyacrylamide gel at 120 V for 60 to 100 min depending on the molecular weight of the proteins. Then, the membranes were blocked with nonfat milk and incubated overnight at 4 °C with antibodies against PI3K (1:1000, Abcam, ab40776, UK), p-PI3K (1:1000, Abcam, ab278545, UK), Akt (1:500, Abcam, ab8805, UK), p-Akt1 (1:1000, Abcam, ab108266, UK), mTOR (1:5000, Abcam, ab134903, UK), p-mTOR (1:1000, Abcam, ab109268, UK), and β-actin (1:2000, Abcam, ab8227, UK). After three washes, the PVDF membranes were incubated with peroxidase-conjugated secondary antibodies (Cell Signaling, 1:1000, USA) for 1 h at room temperature. Finally, the proteins were visualized using the enhanced chemiluminescence method following the manufacturer's instructions (Amersham Biosciences). Quantification was carried out by subtracting the band intensity from the background intensity using ImageJ software (National Institutes of Health, USA). All the Western blot data are presented as protein densitometry/control protein densitometry.

### In vivo animal experiment and surgical procedures

Sixteen female SD rats (200–220 g, eight rats in the PLLA and TCP groups, and eight rats in the 5% and 10% groups) were provided by the Experimental Animal Research Center of Zhejiang Chinese Medical University. All the animal experiments were in accordance with the guidelines established by the local Committee for Animal Experiments. Briefly, an incision was made along the midline of the epicranium. Afterward, a dental drill was used to create two full-thickness standardized round defects (diameter = 5 mm) in symmetry to the sagittal suture. Cylindrical scaffolds (5 mm diameter, 2 mm long) pre-seeded with 5 × 10^4^ PDPCs was inserted in the defect. The periosteum and dermis were closed gently. All the operations were conducted in an aseptic manner. Finally, whole calvaria containing the scaffolds were harvested for evaluation after 4 and 8 weeks of implantation.

### Cell labeling and detection and X-ray examination

Fluorescence signals and X-ray scanning were evaluated by a small animal imaging system (Kodak In-Vivo Imaging System FxPro; Kodak, New York, USA). Before implantation, XenoLight DiR (Caliper Life Sciences, Hopkinton, USA) was used to label the PDPCs for in vivo imaging. The fluorescence signals were detected 4 weeks after implantation to assess the survival of seeded cells. X-ray scanning was carried out to assess the bone regeneration effect of the scaffolds, and the mean gray value of the defect was calculated and then normalized to an unoperated calvarial bone by Image-Pro Plus 6.0.

### Histological examination

The harvested samples at 8 weeks after implantation were fixed in 4% (v/v) neutral buffered paraformaldehyde, decalcified in 10% EDTA solution, and embedded in paraffin blocks. Serial 7-μm-thick sections were obtained from the defect area. Afterwards, the slides were stained with hematoxylin and eosin (H&E) and Masson trichrome staining for new bone assessment. The stained slides were observed by a DP70 CCD camera (Olympus, Tokyo, JAPAN) coupled to an AX-70 microscope (Olympus).

### Immunohistochemistry and quantification

Immunohistochemical staining was performed to analyze OCN, CD206 (macrophage mannose receptor), and inducible nitric oxide synthase (iNOS) expression in the tissue. The primary antibodies were rabbit polyclonal anti-OCN (Sigma-Aldrich, AB10911, USA), rabbit polyclonal anti-CD206 (1:5000, Abcam, ab64693, UK), and rabbit monoclonal anti-iNOS (1:500, Sigma-Aldrich, ZRB1449, USA). The specimens were incubated with the corresponding secondary antibodies conjugated to HRP (Zhongshanjingqiao, Beijing, China). The stained sections were photographed digitally under a microscope.

### Evaluation of local inflammatory responses in vivo rat air pouch model

The assessment of the local inflammatory response for the scaffolds (n = 3) implanted in air pouch after 3 days was carried out by counting the number of inflammatory cells manually. Paraformaldehyde -fixed, paraffin-embedded scaffold samples were cut into consecutive Sects. (7 μm) and stained with H&E and Giemsa in accordance with standard protocols. The number of lymphocytes and neutrophils was counted from randomly selected fields (5 fields per sample) taken at 400 × magnification.

### Statistical analysis

All the data are presented as the mean ± standard deviation. Statistical analyses were carried out using the analysis of variance (ANOVA) method using IBM SPSS Statistics, V.22.0. *P* value < 0.05 was considered a signifcant difference.

## Results

### Identification and characterization of PDPCs

The rat PDPCs maintained a mesenchymal morphology (Additional file 1: Fig. S1A). To evaluate whether the cells possess features of mesenchymal stem cells (MSCs), including clonogenicity and trilineage differentiation potential, colony formation assay were performed and the cells have the excellent ability of colony formation after 2 weeks culture (Additional file 1: Fig. S1B, C). Trilineage differentiation assays identified that PDPCs can differentiate into osteoblasts, chondrocytes, and adipocytes after 3 weeks of induction (Additional file 1: Fig. S1D–F). In addition, these cells expressed a comprehensive series of surface markers, including CD29, CD44 and CD90 (Additional file 1: Fig. S1G–I), which are generally considered to identify PDPCs, while were negative for endothelial, myeloid and hematopoietic markers, such as CD45, CD31 and CD79 (Additional file 1: Fig. S1J–L). Collectively, the outcomes suggested that the PDPCs were successfully isolated.

### Characterization of scaffolds

**Fig. 2:**
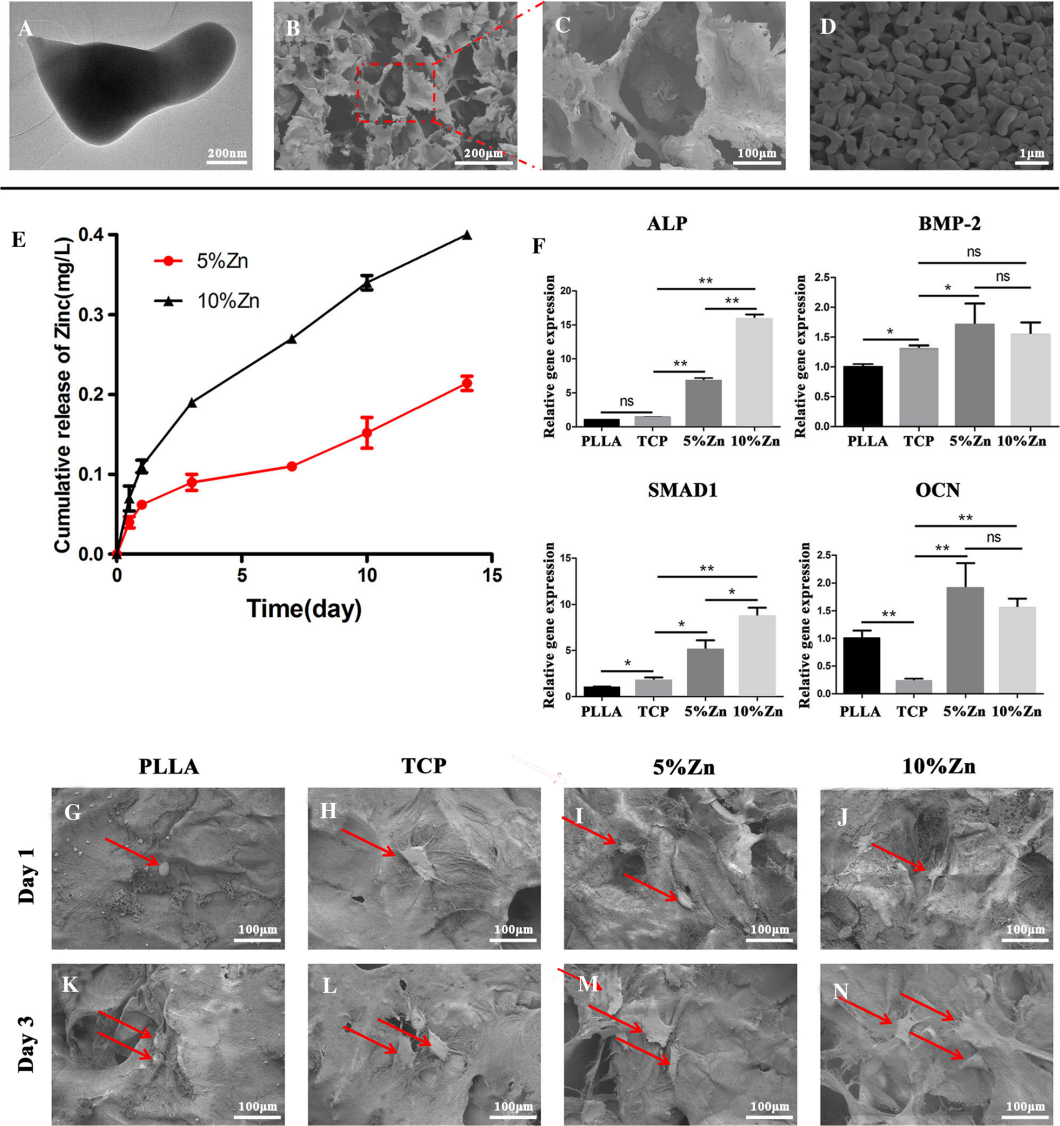
Characterization of Zn-loaded bioactive ceramics and its direct facilitation of the osteogenic differentiation of PDPCs. SEM images of TCP **(A)** and Zn/TCP/PLLA **(B–D)**. **E** The in vitro release curve of Zn^2+^ from Zn/TCP/PLLA scaffolds. **F** Relative mRNA expression levels (normalized to GAPDH) of osteogenesis-related genes (ALP, BMP-2, SMAD1, and OCN) after PDPCs were seeded on the different scaffolds for 7 days. PLLA, PLLA scaffold; TCP, TCP/PLLA scaffold; 5%Zn, 5%Zn-loaded TCP/PLLA scaffold; 10%Zn, 10% Zn-loaded TCP/PLLA scaffold; PDPCs, periosteum-derived progenitor cells;* ns* no significance; *p < 0.05; **p < 0.01. **G–N** SEM images of PDPC-seeded scaffolds (PLLA, TCP/PLLA, 5%Zn/TCP/PLLA, and 10%Zn/TCP/PLLA)

The morphology and microstructure of the Zn-containing TCP particles were detected by TEM. The particles exhibited a dumbbell shape with a size of approximately 200 nm (Fig. 2A). Next, the Zn-containing TCP particles mixed with 5% Pluronic F127 were fabricated into Zn/TCP/PLLA scaffolds. A uniform macropore structure of the scaffold (Zn/TCP particles packaged by PLLA substrates) with a size of approximately 100 μm was identified by SEM (Fig. 2B) and was shown schematically in Additional file 1: Fig. S2A. The TCP particles were observed to randomly agglomerate around the walls of macropores with a size of approximately 50 μm (Fig. 2C, D). The porosities of the TCP, 5%Zn and 10%Zn scaffolds were 91.2%, 86.1% and 85.0%, respectively. Next, the efficacy of the scaffold for Zn^2+^ release was determined. The cumulative release curve suggested that Zn^2+^ delivery was sustained for up to 14 days. There was no initial ‘‘burst’’ release, which might result in adverse effects, in either the 5%Zn or 10%Zn group [5%Zn and 10%Zn were defined as molar ratios of Zn/(Ca + Zn) in the scaffolds] (Fig. 2E). Besides, the Zn^2+^ concentration was far lower as compared with the safe concentration, which was reported to be 5.85 mg/L [41], which meant no potential cytotoxicity to cells. Generally, the Zn^2+^ release from Zn/TCP particles was time and load dependent. More specifically, the Zn content of 10%Zn was observed to be approximately 2 times that of 5%Zn at every time point. Thus, the sustained Zn^2+^-release behavior could be modulated by the Zn content in the Zn/TCP particles. Summarily, both 5%Zn and 10%Zn possess a stable and sustained Zn^2+^-releasing capacity and could be used for all subsequent biological experiments.

### Single effect of scaffolds on the proliferation and osteogenic differentiation of PDPCs

To determine the effect of the scaffolds on the attachment and proliferation of PDPCs, we seeded cells on PLLA, TCP, 5%Zn and 10%Zn (Additional file 2: Fig. S2A), observed the morphology by SEM for 1 day and 3 days and detected cell vitality and proliferation by live/dead staining and CCK-8 assay, respectively. The SEM images identified that the PDPCs had successfully attached to the scaffolds, and displayed spherical shapes after 1 day (Fig. 2G–J). Furthermore, 3 days after being seeded, the cells gradually proliferated, spread well with pseudopodia anchoring onto the scaffold surfaces, and displayed a dendritic appearance, particularly on the 5%Zn and 10%Zn surfaces (Fig. 2K–N). Live/dead staining (Additional file 3: Fig. S3A) and CCK-8 (Additional file 3: Fig. S3B) showed that cells seeded in all groups proliferated over time with a high number of live cells, suggesting that 5%Zn and 10%Zn are potentially suitable for in vivo applications.

To evaluate the osteoinduction capacity of the four types of scaffolds on PDPCs, we analyzed the mRNA expression levels of osteogenic genes, including ALP, BMP-2, Smad1, and OCN, after 7 days of culture on the scaffolds (Fig. 2F). The expression levels of BMP-2 and OCN genes were upregulated in the 5%Zn and 10%Zn groups, with no significant difference between different Zn doses. However, the expression levels of the ALP and Smad1 genes were dose-dependently upregulated. The above data suggested that the 10%Zn-loaded scaffold possessed the best capacity for spontaneously inducing the osteogenic differentiation of PDPCs.

### Immunomodulatory effects of scaffolds

**Fig. 3:**
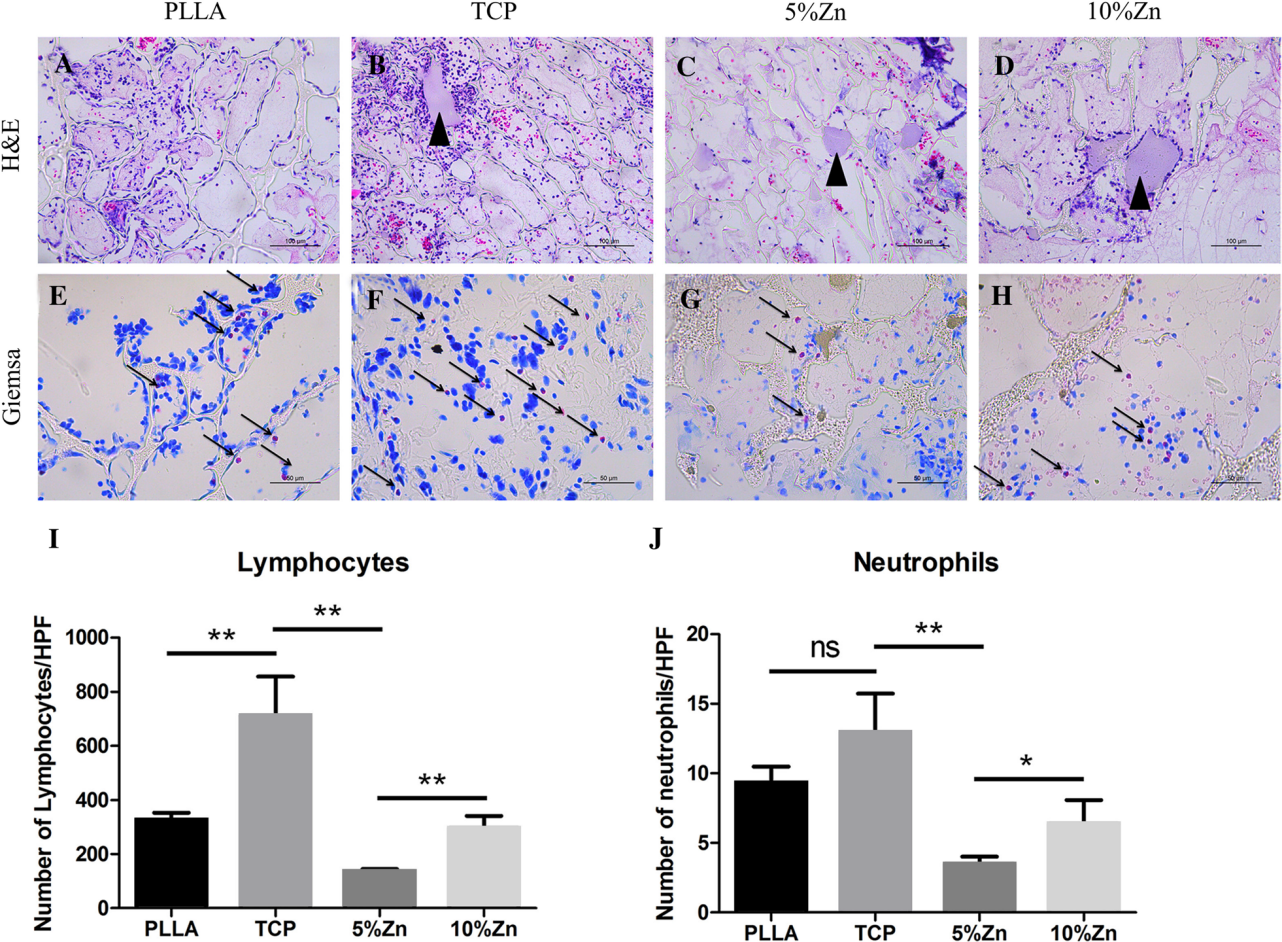
The moderate local inflammatory reaction elicited by scaffolds at the beginning of grafting in vivo. H&E (scale bar = 100 μm) **(A–D)** and Giemsa (scale bar = 50 μm) **(E–H)** staining of the scaffolds implanted subcutaneously in rats for 3 days, respectively. Statistical semiquantification of lymphocyte **(I)** and neutrophil **(N)** invasion of histological sections from the implanted scaffolds. PLLA, PLLA scaffold; TCP, TCP/PLLA scaffold; 5%Zn, 5%Zn-loaded TCP/PLLA scaffold; 10%Zn, 10%Zn-loaded TCP/PLLA scaffold; ns, no significance; *p < 0.05; **p < 0.01

As local immunity has recently been demonstrated to play a central role in bone regeneration, we evaluated the immunomodulatory effects of scaffolds in vivo and in vitro. First, scaffolds were implanted subcutaneously (air pouch model) in rats for 3 days, followed by histological evaluation of inflammatory cells. Significantly less lymphocyte and neutrophil invasion was observed in the 5%Zn and 10%Zn samples than in the PLLA and TCP samples, indicating a relatively moderate inflammatory reaction for Zn-loaded scaffolds at the beginning of scaffold grafting (Fig. 3A–J).

It is universally accepted that lymphocytes and neutrophils predominate in the early stage after scaffold implantation and are then gradually replaced by monocytes/M$\varphi$s [42]. Moreover, the phenotypes and functions of monocyte-derived M$\varphi$s were reported to play a regulatory function in the bone repair process [36]. Thus, we explored the effect of scaffolds on the proliferation and functional phenotypes of M$\varphi$s. RAW264.7 cells were seeded on PLLA, TCP, 5%Zn and 10%Zn in vitro (Additional file 2: Fig. S2B). Similarly, the CCK-8 assay (Additional file 3: Fig. S3C) and live/dead staining (Additional file 3: Fig. S3D) suggested that none of the four scaffolds had cytotoxic effects on the RAW264.7 cells.

**Fig. 4:**
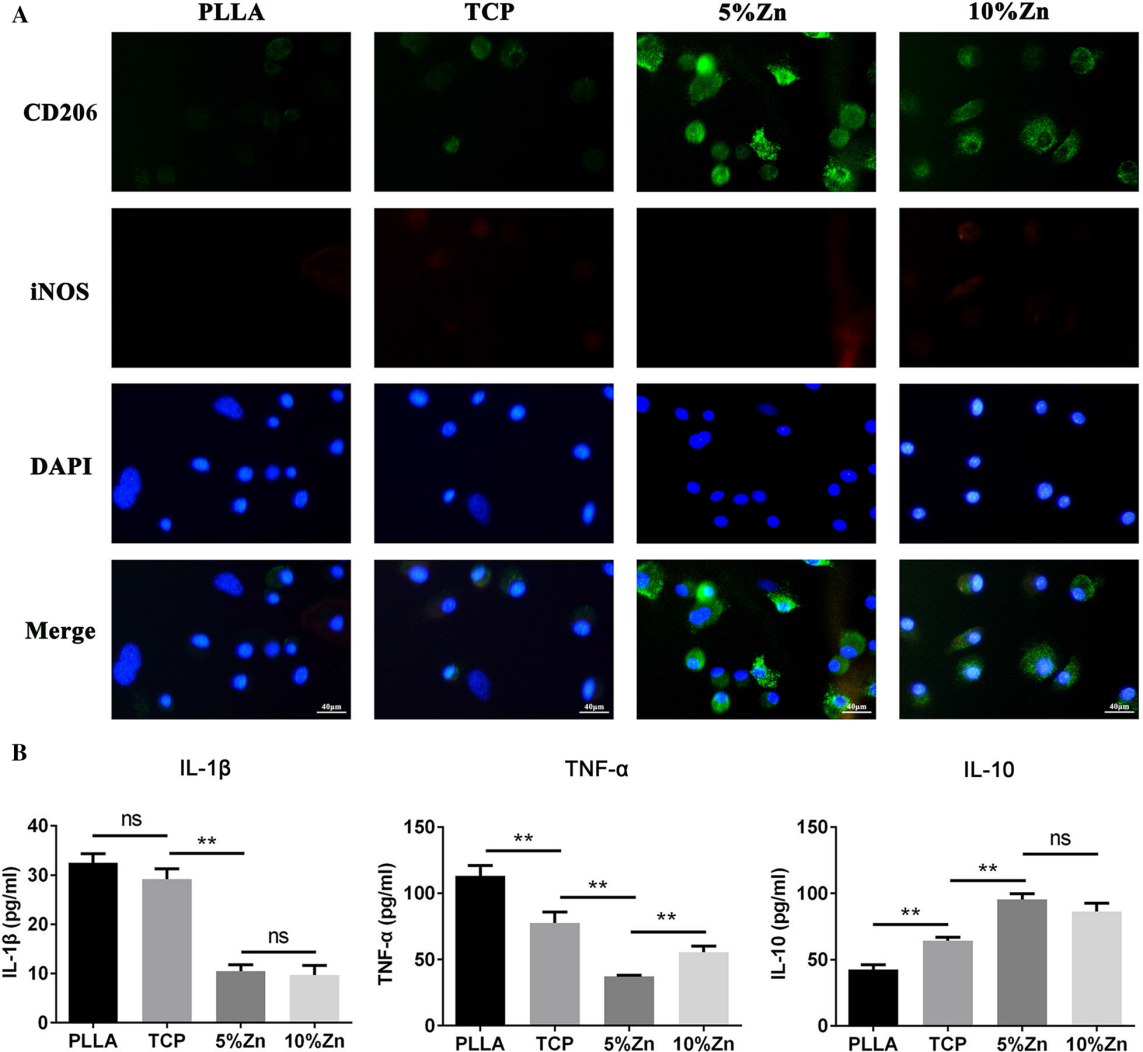
The effects of Zn-loaded bioactive ceramics in promoting the M2 polarization of M$\varphi$s. **A** Immunofluorescence staining images of M$\varphi$s seeded on scaffolds for 3 days. CD206 (green) and iNOS (red), DAPI (blue). Scale bar = 40 μm. **B** The levels of the cytokines IL-1β, TNF-α, and IL-10 in the culture medium of M$\varphi$s seeded on scaffolds for 3 days were analyzed by ELISA. PLLA, PLLA scaffold; TCP, TCP/PLLA scaffold; 5%Zn, 5%Zn-loaded TCP/PLLA scaffold; 10%Zn, 10%Zn-loaded TCP/PLLA scaffold; M$\varphi$ macrophages;* ns*no significance; *p < 0.05; **p < 0.01

Next, the polarity of M$\varphi$s was subsequently investigated by immunofluorescence and ELISA. Notably, much higher levels of the M2 marker CD206 were expressed in M$\varphi$s on Zn-loaded scaffolds than on other scaffolds, especially on 5%Zn (Fig. 4A). Consistent with this, the anti-inflammatory factor IL-10, which is also recognized as an M2 marker, was markedly increased in the culture medium, while the expression levels of the proinflammatory factors IL-1β and TNF-α, which are usually considered M1 markers, were greatly downregulated in the Zn-loaded groups (Fig. 4B). These data suggested that Zn-loaded scaffolds, especially 5%Zn, could induce the M2 polarization of M$\varphi$s.

**Fig. 5:**
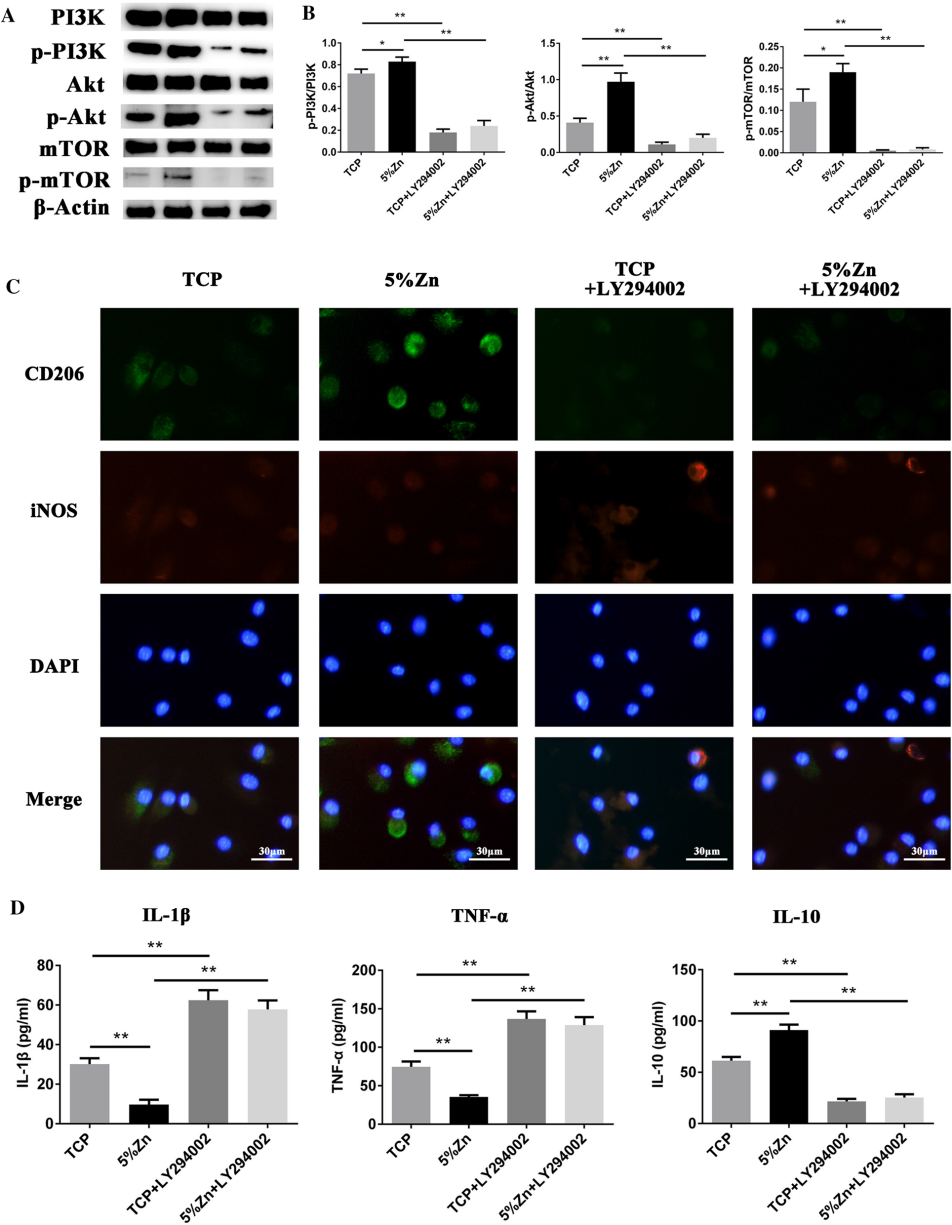
The PI3K/Akt/mTOR pathway was involved in Zn-loaded scaffolds-induced M2 polarization of M$\varphi$s. M$\varphi$s were incorporated into TCP/PLLA and 5%Zn/TCP/PLLA for 3 days with or without the PI3K inhibitor LY-294002 (40 μM). **A** The protein levels of PI3K, p-PI3K, Akt, p-Akt, mTOR, p-mTOR, and β-actin were analyzed by Western blotting. The protein bands from left to right was TCP, 5%Zn, TCP + LY294002, and 5%Zn + LY294002, respectively. **B** Quantitative analysis of Western blots showing the p-PI3K/PI3K, p-Akt/Akt, and p-mTOR/mTOR ratios. **C** The levels of IL-1β, TNF-α, and IL-10 cytokines in the culture medium of M$\varphi$s were analyzed by ELISA. **D** Immunofluorescence staining images of M$\varphi$s after treatment for 3 days. CD206 (green) and iNOS (red), DAPI (blue). Scale bar = 40 μm. M$\varphi$macrophages; ns, no significance; *p < 0.05; **p < 0.01

To further investigate the internal mechanism of Zn^2+^-driven M$\varphi$ polarization, M$\varphi$s were incorporated onto TCP/PLLA and 5%Zn/TCP/PLLA with or without the PI3K inhibitor LY-294002 (40 μM). The levels of p-PI3K, PI3K, Akt, p-Akt, mTOR, and p-mTOR were detected by Western blotting. As shown in Fig. 5A, B, Zn-loaded TCP/PLLA significantly increased the ratios of p-PI3K/PI3K, p-Akt/Akt and p-mTOR/mTOR, indicating activation of the PI3K/Akt/mTOR pathway. Moreover, the expression of M2 M$\varphi$-associated markers (CD206 and IL-10) also significantly increased in the 5%Zn/TCP/PLLA groups compared with the TCP/PLLA groups, and this phenomenon could be largely rescued after LY294002 administration (Fig. 5C, D). This evidence indicated that Zn-loaded scaffolds induced M2-polarized M$\varphi$s largely through PI3K/Akt/mTOR signaling.

### Single effect of scaffolds induced directional polarization of M$\varphi$s on PDPC osteogenesis in 2D cultures

**Fig. 6:**
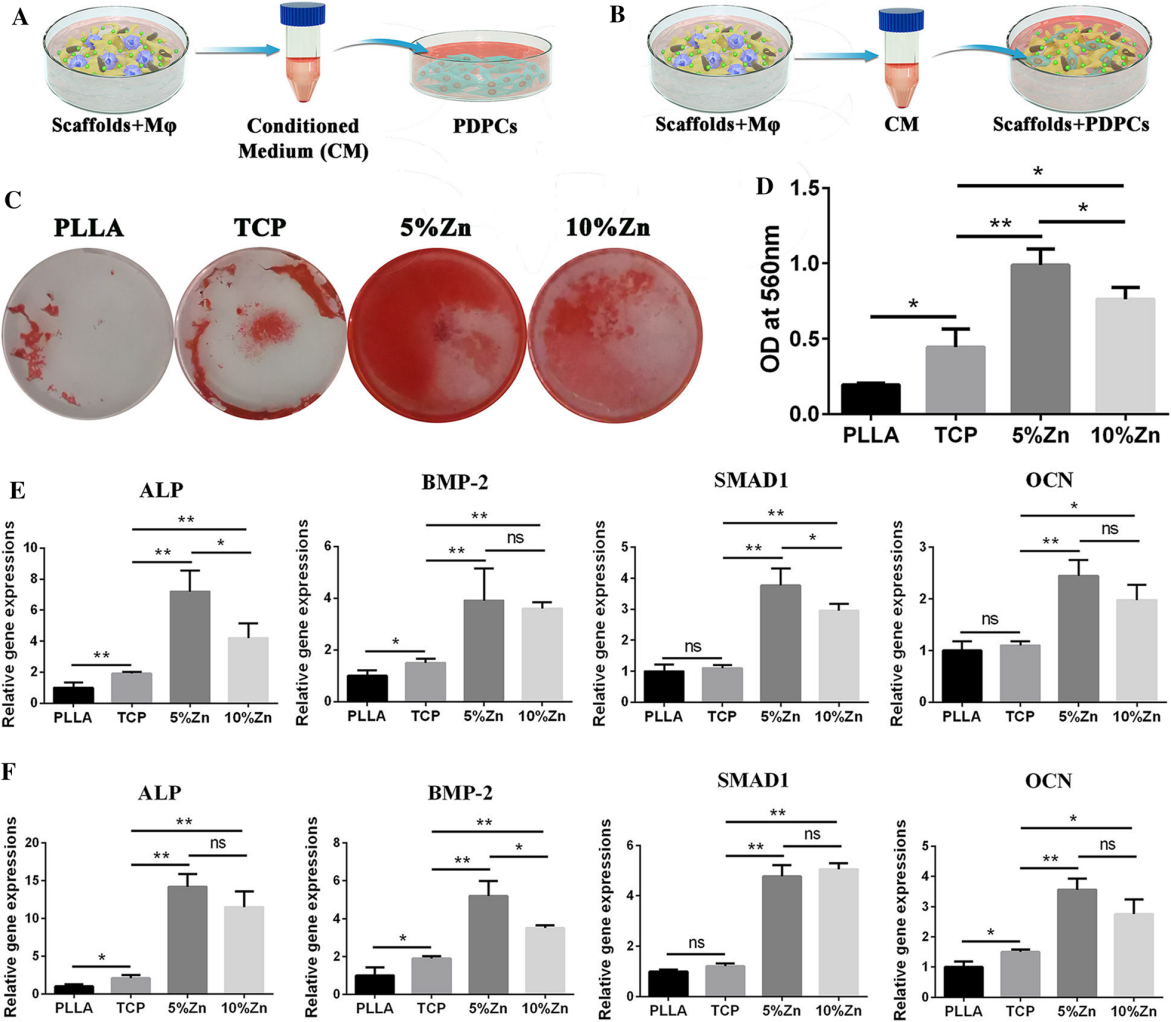
Reciprocal interactions between osteoinductive and immunomodulatory characteristics of the Zn-loaded bioactive CaP scaffolds. **A** Schematic illustration of the effect of CM from scaffold-induced polarized M$\varphi$s on the osteogenic differentiation of PDPCs. **B** Schematic illustration of the effect of CM from scaffold-induced polarized M$\varphi$s on the osteogenic differentiation of PDPCs seeded on the corresponding scaffold. **C** Alizarin red staining and **(D)** semiquantification of mineralized nodules for PDPCs, as illustrated in (**A**). **E** Relative mRNA expression levels (normalized to GAPDH) of osteogenesis-related genes (ALP, BMP-2, SMAD1, and OCN) in PDPCs, as illustrated in (**A**). **F** Relative mRNA expression levels (normalized to GAPDH) of osteogenesis-related genes (ALP, BMP-2, SMAD1, and OCN) in PDPCs, as illustrated in (**B**). PLLA, PLLA scaffold; TCP, TCP/PLLA scaffold; 5%Zn, 5%Zn-loaded TCP/PLLA scaffold; 10%Zn, 10%Zn-loaded TCP/PLLA scaffold; PDPCs, periosteum-derived progenitor cells;* ns* no significance, M$\varphi$ macrophages, *CM* conditioned medium; *ns* no significance; *p < 0.05; **p < 0.01

To evaluate the immunomodulatory osteogenic effect of the samples, we collected supernatants from RAW264.7-seeded scaffolds and prepared M$\varphi$-conditioned medium. PDPCs in cell culture plates were then cultured with M$\varphi$-conditioned medium to assess their osteogenic differentiation ability (Fig. 6A). After 14 days of culture, Alizarin Red S staining of PDPCs was performed to determine the immune-related osteogenic effects. The outcomes suggested that the M$\varphi$-conditioned medium derived from the 5%Zn group had the most mineralized nodule formation and the highest calcium deposition, which indicated the best osteogenic effect compared with the other groups (Fig. 6C, D). Accordingly, a significantly higher mRNA expression level of osteogenic genes, including ALP, BMP-2, Smad1, and OCN, was also exhibited in the 5%Zn group than in the other groups after 7 days of culture (Fig. 6E). These outcomes showed that M$\varphi$s cultured on 5%Zn-loaded scaffolds secreted a series of M2-related cytokines, which possessed the best immunomodulatory osteogenic effect.

### Synergistic effect of scaffolds and scaffold-induced directional polarization of M$\varphi$s on PDPC osteogenesis

To determine the synergistic effect of the scaffolds and scaffold-induced directional polarization of M$\varphi$s on osteogenic differentiation, PDPCs were seeded on scaffolds and cultured with the corresponding scaffold-induced M$\varphi$-conditioned medium (Fig. 6B). After 7 days of culture, genes associated with osteogenesis were upregulated in Zn-loaded scaffolds, especially in 5%Zn, as expected (Fig. 6F). In summary, the combination of Zn-loaded scaffolds and the local scaffold-induced immune microenvironment interacted to enhance stem cell osteogenic differentiation.

### In vivo bone regeneration with PDPCs loaded scaffolds

**Fig. 7:**
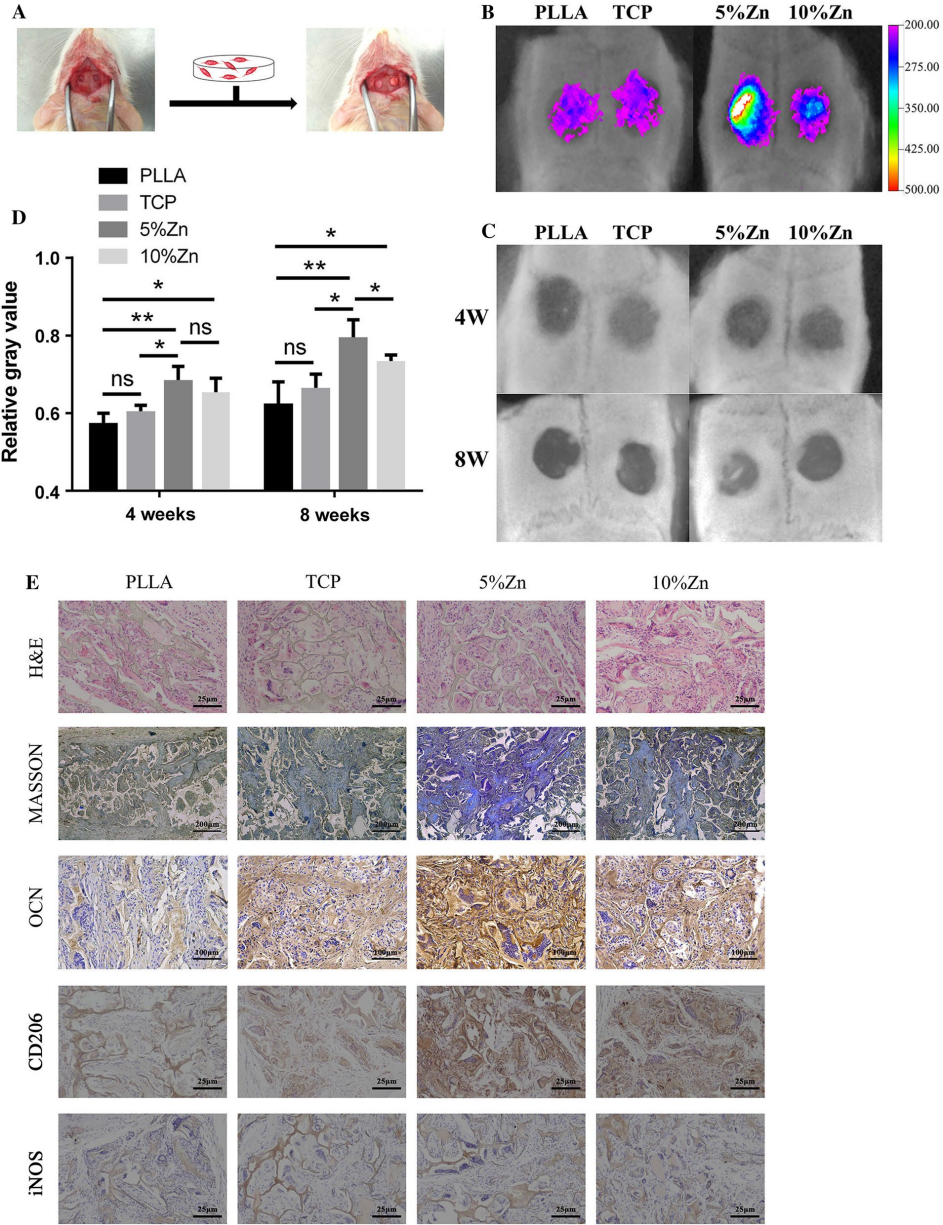
Endogenous bone regeneration in a calvarial critical-size defect model by PDPCs incorporated Zn-loaded bioactive ceramics. **A** Schematic diagram of a bone defect and implantation. **B** Detection of the fluorescence signal emitted by DiR at 4 weeks postimplantation. **C** X-ray scanning and **D** the mean gray value calculation of defects at 4 and 8 weeks postimplantation. **E** Histological characterization of tissue at the defect site at 8 weeks postimplantation. PLLA, PLLA scaffold; TCP, TCP/PLLA scaffold; 5%Zn, 5%Zn-loaded TCP/PLLA scaffold; 10%Zn, 10%Zn-loaded TCP/PLLA scaffold;* ns* no significance; M$\varphi$, macrophages; PDPCs, periosteum-derived progenitor cells;* ns* no significance; *p < 0.05; **p < 0.01

To evaluate the capability of Zn-containing TCP/PLLA to promote bone growth in vivo, we created critical-size cranial bone defects in SD rats and implanted PDPC-loaded scaffolds labeled with DiR (Fig. 7A). Detection of the signal emitted by DiR after 4 weeks showed that labeled PDPCs participated in bone regeneration, and the signal in the 5%Zn group was much stronger than that in the other groups, indicating a higher ratio of surviving PDPCs (Fig. 7B). These results demonstrated that a local immune microenvironment conducive to PDPC survival and proliferation was established by 5%Zn.

Next, the bone regeneration effects were assessed by X-ray in a critical calvarial defects model after scaffold implantation for 4 and 8 weeks (Fig. 7C). More radiopaque tissues was observed in the Zn-containing TCP/PLLA groups, particularly in the 5%Zn group (Fig. 7C). Quantification of radiopaque tissues in the defect area by calculating the relative gray value demostrated that the grey value in the 5%Zn group was statistically elevated compared with that in the other groups (*P* < 0.05) at 8 weeks (Fig. 7D). Moreover, histological examination was conducted to evaluate new bone growth in defect areas (Fig. 7E). Newly formed bone, collagenous tissues and implanted scaffolds were clearly identified in the H&E- and Masson-stained images. A pronounced bone formation process was observed in the Zn-containing scaffolds, especially in 5%Zn, which was predominantly filled with abundant fibrous tissue (Fig. 7E). Consistently, the highest expression of the osteogenic marker OPN was found in the 5%Zn-loaded scaffold (Fig. 7E). In addition, cell morphology was maintained in organs, including the heart, liver, spleen, lungs, and kidneys, in all of the implant groups, indicating biocompatibility and biosafety with tissue in vivo (Additional file 4: Fig. S4).

Next, the capacity of Zn-containing scaffolds to recruit host M$\varphi$s and induce their directional polarization was further confirmed by immunohistochemistry. Similar to the in vitro results, a markedly increased number of CD206-positive cells was observed in the 5%Zn group, while there was no significant difference in the number of cells positive for the M1 marker iNOS among the groups (Fig. 7E). Thus, 5%Zn-loaded scaffolds could effectively induce M2 polarization of host M$\varphi$s in vivo, which plays a critical role in the immunomodulatory osteogenesis of the loaded PDPCs.

## Discussion

In the present study, we fabricated Zn-loaded bioactive ceramics and analyzed the synergistic effect of the scaffold and scaffold-induced directional polarization of M$\varphi$s on PDPC osteogenesis in vitro and in vivo. Our results indicated that this scaffold could not only directly promote PDPC osteogenic differentiation but also induce M2 polarization of M$\varphi$s and subsequently create a favorable osteogenic microenvironment for PDPCs.

Due to limitations in reconstructing critical bone defects, developing bioactive scaffolds for bone tissue engineering has become an alternative strategy for skeletal reconstruction [20, 43]. Among all these scaffolds, synthetic bioactive ceramics, such as hydroxyapatite, β-TCP, calcium polyphosphate, and biphasic calcium phosphate, are widely used as basic bone repair materials due to their excellent osteoconductive features and controllable chemical reagent deposition process [44]. Although some osteogenic biomacromolecules, such as recombinant human bone morphogenetic protein-2 (rhBMP-2), have been successfully loaded into these ceramics, serious side effects associated with ectopic or unwanted bone formation in certain situations have caused the Food and Drug Administration (FDA) to be increasingly cautious in approving the application of such materials [45, 46].

A potentially safer approach is the incorporation of some trace metallic ions, such as Zn^2+^, Mg^2+^, Sr^2+^, Cu^2+^ and Co^2+^, into these bioactive ceramics [47]. Among these trace metallic ions, Zn^2+^ is an ion of high interest due to its indispensable role in the natural bone remodeling process and bone diseases [48, 49]. For example, Zn^2+^ is an indispensable cofactor for ALP during the bone mineralization process [50]. Dietary Zn deficiency is closely related to delayed skeletal development and defective bone regeneration in humans and animals [50]. Hence, several studies have incorporated Zn into bioactive ceramics for bone tissue engineering, and Zn-eluting hydroxyapatite composites have been observed to improve the osteogenic induction of MSCs [51–55]. Similarly, studies have confirmed that Zn-containing TCP composites promote the osteogenic differentiation of MSCs [56–58]. Additionally, moderate Zn-loaded calcium phosphate cements greatly contributed to the improvement of the ALP activity of MSCs [59]. The effect of Zn-loaded bioactive ceramics on the osteogenesis lineage-specific differentiation of MSCs has been fully investigated, and favorable outcomes have been observed.

However, few studies have focused on the immune response of the host to these Zn-loaded bioactive ceramics and the cellular crosstalk between MSCs and the biomaterial-mediated immune response; innate immune cells, particularly M$\varphi$s, are main constituents of the immune system and interact with MSCs to powerfully direct bone healing [36, 60]. Luo et al. investigated the effects of different doses of Zn-loaded TCP on the osteoclastogenic differentiation of RAW264.7 cells [57]. The presence of osteoclasts might be positive and indirectly indicate the number of M1-polarized M$\varphi$s, which are commonly considered the precursor state of osteoclasts [61]. This outcome suggested that Zn^2+^, but not the substrate TCP, predominantly determined M$\varphi$ fate, and only a high concentration released of Zn^2+^ (as high as 1.8 ppm) from TCP could activate the osteoclastic differentiation induction capacity of the M$\varphi$s. Similarly, another study identified that a low concentration of Zn^2+^ (less than 6.5 ppm) had no effect on osteoclast activity, while higher Zn^2+^ amounts increased the number of TRAP-positive cells [62]. These results presented herein are consistent with our findings that both 5% Zn- and 10% Zn-loaded TCP/PLLA scaffolds had no effect on the polarization of M1 M$\varphi$s (Fig. 4A). Zn^2+^ was released from our scaffolds at 0.4 mg/l (i.e., 0.40 ppm, as shown in Fig. 2E) at 14 days, which is much lower than 6.5 ppm. However, to the best of our knowledge, no study has directly identified the regulatory effect of Zn-loaded ceramics on the polarization of M$\varphi$s and the ensuing osteoimmunomodulation.

In the present study, the outcomes suggested that Zn/TCP/PLLA could effectively promote the M2 polarization of M$\varphi$s. More specifically, TCP/PLLA weakly facilitated the polarization of M$\varphi$s toward the M2 phenotype, while both 5% and 10%Zn/TCP/PLLA were found to be strong driving forces (Fig. 4). Recently, studies have explored the inconsistent effects of TCP on M$\varphi$ phenotypes. Jia et al. [33] explored that TCP could shift M$\varphi$ polarization towards the M2 phenotype both in vitro and in vivo. Chen et al. [34] and Zheng et al. [63] showed that the phenotype of M$\varphi$s switched to M2 in response to β-TCP extracts. Gu et al. [64, 65] explored whether introducing a certain amount of β-TCP into calcium phosphate cement could promote M$\varphi$ polarization to the anti-inflammatory phenotype (M2) in long-term culture. In contrast, Tai et al. [66], Chen et al. [67], and Fernandes et al. [68] found that β-TCP, its extract, and its use as a coating on a Ti surface were strong enough to induce M1 polarization of M$\varphi$s. Chen et al. [69] also identified that β-TCP maintained a high proportion of iNOS^+^ M1-polarized M$\varphi$s both in vitro and in vivo. In our study, TCP was wrapped in PLLA to form a porous structure. Only a small amount of TCP is in direct contact with the culture medium/bodily fluid and releases calcium and phosphate ions to modulate the M$\varphi$ phenotype. Thus, this could explain the relatively weak effect of the TCP/PLLA scaffold in the regulation of M$\varphi$ polarization in short-term culture. As a biodegradable polymer, PLLA gradually degrades to form increased numbers of caverns, which enable an increased exposure of β-TCP for ion exchange. Therefore, the regulatory effect of β-TCP on host M$\varphi$s might accelerate gradually in long-term implantation.

Over the years, Zn element has been extensively focused on in the field of bone tissue engineering because of its excellent antibacterial [70, 71], antioxidant and anti-inflammatory [72, 73] and direct osteogenic effects [50, 71]. Research on the anti-inflammatory properties of Zn^2+^ has mainly focused on the reduced release of inflammatory factors and decreased immune cell infiltration [74–78]. Recent studies have shifted from the viewpoint that biomaterials should be designed to minimize the host inflammatory response to the concept that biomaterials should play an immunomodulatory role during the bone regeneration process [20, 79]. Therefore, the potential of Zn^2+^ loaded scaffolds to regulate M$\varphi$s polarization for tissue engineering has been gradually explored (Table 2). Zhu et al. [28] and Chen et al. [29] observed that Zn-decorated Ti/TiO_2_ surfaces induced the M2 state of M$\varphi$s and had better osseointegration or bone regeneration capacity. Additionally, Zn-loaded silica-nanofibrous polymers attained the lowest M1/M2 ratio of M$\varphi$s and achieved the greatest efficiency for bone and vascular regeneration [30]. In addition, a Zn-modified sulfonated polyetheretherketone surface was observed to modulate inactivated M$\varphi$ polarization to an anti-inflammatory phenotype and promote the secretion of anti-inflammatory and osteogenic cytokines [80]. Besides, Zn-doped microcrystalline bioactive glass was observed to induce M$\varphi$s’ sequential transition from M1 to M2, which facilitated bone regeneration [81]. Also, ZnCl_2_-doped sol–gel coating could induce an increase of IL-1β, TGF-β, and IL-4 gene expression in M$\varphi$s [82]. However, another study reported that both Zn^2+^ supplementation (50 µM) and deficiency promoted M1 polarization of THP-1-derived M$\varphi$s [83]. This inconsistency might be attributed to the lower concentration of Zn^2+^, the shorter incubation time (6 h and 24 h) or the different M$\varphi$ type used in the study than in the others. In summary, most of the outcomes were consistent with our findings that Zn decoration could markedly induce M2 polarization.

**Table 2:** Summarized studies investigating the role of Zn-loaded scaffolds on M$\varphi$'s biological behavior for bone tissue

M$\varphi$s origin	Type of Zn loaded scaffolds	Effect on M$\varphi$s	Functions	Reference(s)
RAW264.7	Zn-decorated Ti surfaces	Inhibit adhesion and proliferation; induce M2 states	Osseointegration	[28]
RAW 264.7	Zn-incorporated TiO_2_ Nanotube	Promote M2 markers; Moderately inhibit M1 markers	Bone formation	[29]
In vivo^a^	Zn loaded silica-nanofibrous polymers	Attain the lower ratio M1/M2	Bone regeneration	[30]
RAW264.7	Zn-containing tricalcium phosphate	Induce proliferation and osteoclastogenesis	Bone regeneration	[57]
RAW 264.7	Zn-modified sulfonated polyetheretherketone	Polarize M$\varphi$ to an anti-inflammatory phenotype	Bone regeneration	[80]
THP-1	Zn-doped porous microcrystalline bioactive glass	Induce sequential M1-to-M2 transition	Bone regeneration	[81]
RAW264.7	ZnCl_2_-doped sol–gel coating	Induce anti-inflammatory markers; reduce inflammatory markers	Bone regeneration	[82]

*Zn* zinc, M$\varphi$ macrophage

^a^M$\varphi$ in New Zealand white rabbits

To the best of our knowledge, few studies have elucidated the exact molecular mechanisms underlying Zn-driven M$\varphi$ polarization. It has been widely reported that Zn^2+^ can activate PI3K/Akt/mTOR signaling in various tissue types [84–90]. Given that the PI3K/Akt pathway significantly affects the polarization phenotype of M$\varphi$s [91, 92], we sought to determine whether this signaling plays a role in Zn-induced M2 polarization of M$\varphi$s. Our data showed that the addition of Zn to the scaffold significantly activated the PI3K/Akt/mTOR pathway, which was accompanied with an increased number of M2-polarized M$\varphi$s, while these effects could be largely eliminated by the PI3K inhibitor LY294002. Thus, the scaffold containing Zn induced the M2 polarization of M$\varphi$s largely through the PI3K/Akt/mTOR pathway.

In the present study, the osteogenic effect of the Zn loading CaP scaffolds were comprehensively identified by three steps: First, the scaffolds could directly induce the osteogenic differentiation of PDPCs (single osteogenic effect of the scaffolds on stem cells). Second, the Zn loading CaP scaffolds could trigger a pro-healing immune stimuli (M2 polarized macrophages), which subsequently promoted osteogenesis of PDPCs (single immunomodulatory osteogenic effect of the scaffolds). Third, the synergistic effects of the scaffolds and pro-healing immune stimuli on bone regeneration were observed through seeding PDPCs on scaffolds and culturing them with the corresponding scaffold-induced M$\varphi$-conditioned medium (the synergistic effects of the scaffolds and the local scaffold-induced immune microenvironment on bone regeneration). Interestingly, 10%Zn-loaded TCP/PLLA possessed the best capacity to spontaneously promote osteogenic differentiation of PDPCs, while 5%Zn-loaded TCP/PLLA demonstrated the strongest ability to modulate the M2 polarization of M$\varphi$s, which subsequently created the best immunomodulatory osteogenic microenvironment for PDPCs. It is worth comprehensively comparing the synergistic osteogenic effect of 5% and 10%Zn/TCP/PLLA, considering the effects of both the scaffold itself and the local immune microenvironment induced by the scaffold. The outcomes indicated that 5%Zn-loaded TCP/PLLA had a much more robust synergistic effect on PDPC osteogenesis than 10%Zn/TCP/PLLA. This phenomenon provided straightforward evidence that the influence of the microenvironment created by scaffolds overwhelmed the benefits of the scaffold itself to some extent. In the current study, the effects of the immune microenvironment on PDPCs were indirectly explored by adding conditioned medium from M$\varphi$s grown on scaffolds to PDPC-loaded scaffolds. Strictly speaking, inoculation and examination of direct MSC-M$\varphi$ contact in the same scaffold would have been more persuasive in the current study. However, it might be frustratingly difficult to individually detect the levels of osteoblast-related genes (such as ALP, BMP-2, Smad1, and OCN) in PDPCs in a mixed cell system. Moreover, the source of M$\varphi$s in the study, RAW 264.7 cells, proliferate rapidly and infinitely. If PDPCs and RAW 264.7 cells were cocultured directly in the same scaffold, RAW 264.7 cells would rapidly invade the entire scaffold.

It should be noted that the local immune response after biomaterial implantation may not be restricted to M$\varphi$ activation. A previous study showed that silicified collagen biomaterials induced osteogenesis and angiogenesis through monocyte immunomodulation [93]. In addition, another study showed that fibrinogen scaffolds promoted in vivo bone regeneration by regulating the proportions of surrounding systemic immune cell populations, including T, B, NK and NKT lymphocytes and myeloid cells [94]. This work demonstrates that Zn/TCP/PLLA promotes the M2 polarization of host M$\varphi$s and creates a favorable osteoimmune microenvironment, thus providing insight into biomaterial-guided endogenous bone regeneration. Future studies will focus on other types of immune cells, such as T, B, NK and NKT lymphocytes and myeloid cells, in the adaptive response and their contributions to Zn/TCP/PLLA-guided bone regeneration processes.

## Conclusion

The immunomodulatory osteoinductive effects of the Zn-loaded bioactive ceramics were fully elucidated in the present study. We showed that this scaffold played a dual role in endogenous bone regeneration by directly inducing PDPC osteogenic differentiation and by promoting M$\varphi$ towards a pro-healing phenotype (M2), which subsequently further enhanced PDPC-mediated bone formation. These findings could provide a better understanding of the role of the host immune system on the osteogenic effect of Zn-loaded bioactive ceramics and will demonstrate a novel concept of coupling spontaneous osteogenesis with favorable osteoimmunomodulation scaffold implantation strategies for bone regeneration.

## Supplementary Information

**Additional file 1: Figure S1.** Evaluation of PDPC stemness and cell markers.** A** Representative images of PDPCs under an optical microscope. scale bar = 300 μm. **B, C** Representative images of colonies formed by PDPCs after 2 weeks of culture. **D–F** The ability of PDPCs to differentiate into osteogenic, chondrogenic, and adipogenic lineages assessed by Oil Red O staining (scale bar = 100 μm), Alizarin red staining (scale bar = 100 μm), and Safranin O staining (scale bar = 100 μm), respectively. **G–L** Flow cytometric analysis identified cell surface markers (CD29, CD44, CD90, CD31, CD45, and CD79). PDPCs, periosteum-derived progenitor cells.

**Additional file 2: Figure S2.** Schematic illustration of PDPC-seeded **A** and M$\varphi$-seeded **B** scaffolds (PLLA, TCP/PLLA, 5%Zn/TCP/PLLA, and 10%Zn/TCP/PLLA)

**Additional file 3: Figure S3. **Influence of scaffolds on cytotoxicity and proliferation of incorporated PDPCs and M$\varphi$s. Live/dead staining of PDPCs **(A)** and M$\varphi$s **(D)** incorporated in scaffolds on days 6 and 3, respectively. Living cells were stained with calcein AM (green fluorescence), and dead cells were stained with PI (red fluorescence). Scale bar = 40 μm. Proliferation of PDPCs **(B)** and M$\varphi$s **(C)** in scaffolds as determined by CCK-8 assays during 6-day and 3-day incubation periods, respectively. PDPCs, periosteum-derived progenitor cells; M$\varphi$ macrophages,* ns* no significance; *p < 0.05; **p < 0.01.

**Additional file 4: Figure S4. **Toxicity evaluation of scaffolds in vivo. Tissue sections of the heart, liver, spleen, lung and kidney from rats treated with scaffolds were analyzed via H&E staining. Scale bar = 100 μm.

## Data Availability

The data used to support the findings of this study are available from the corresponding author upon request.

## Abbreviations

ALP: Alkaline phosphatase
ANOVA: Analysis of variance
BMP-2: Bone morphogenetic protein-2
BMSCs: Bone marrow stem cells
CCK-8: Cell Counting Kit-8
CD206: Macrophage mannose receptor
DAPI: 4′,6‐Diamidino‐2‐phenylindole
DMEM: Dulbecco’s Modified Eagle’s Medium
ELISA: Enzyme-linked immunosorbent assay
FBS: Fetal bovine serum
FDA: Food and Drug Administration
FITC: Fluorescein isothiocyanate
H&E: Hematoxylin and eosin
iNOS: Inducible nitric oxide synthase
M$$\varphi $$: Macrophage
OCN: Osteocalcin
PBS: Phosphate-buffered saline
PDPCs: Periosteum-derived progenitor cells
PE: R-phycoerythrin
PLLA: Poly(L-lactic acid)
rhBMP-2: Recombinant human bone morphogenetic protein-2
SEM: Scanning electron microscopy
TCP: Tricalcium phosphate
Zn: Zinc
Zn^2^^+^: Znic ion
β-TCP: β-Tricalcium phosphate
