# Supplementary figures and images for: Sustained zinc release in cooperation with CaP scaffold promoted bone regeneration via directing stem cell fate and triggering a pro-healing immune stimuli
Source: J Nanobiotechnology. 2021 Jul 12;19:207. doi: 10.1186/s12951-021-00956-8 (PMC8274038; doi:10.1186/s12951-021-00956-8)

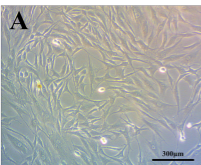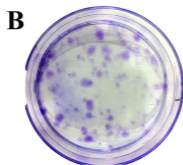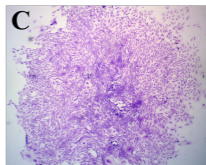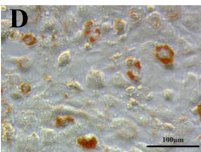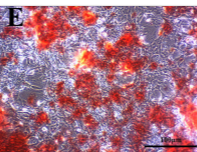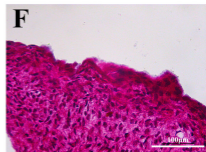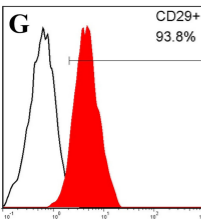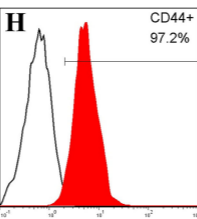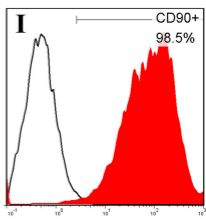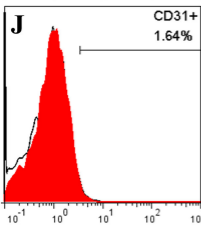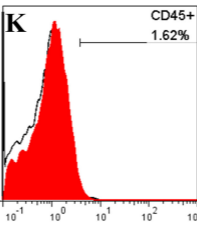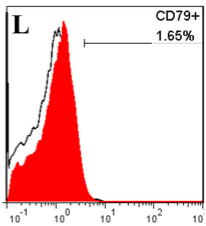

Supplement: Supplementary file 1 — Additional file 1: Figure S1. Evaluation of PDPC stemness and cell markers. A Representative images of PDPCs under an optical microscope. scale bar = 300 μm. B, C Representative images of colonies formed by PDPCs after 2 weeks of culture. D–F The ability of PDPCs to differentiate into osteogenic, chondrogenic, and adipogenic lineages assessed by Oil Red O staining (scale bar = 100 μm), Alizarin red staining (scale bar = 100 μm), and Safranin O staining (scale bar = 100 μm), respectively. G–L Flow cytometric analysis identified cell surface markers (CD29, CD44, CD90, CD31, CD45, and CD79). PDPCs, periosteum-derived progenitor cells. [file 12951_2021_956_MOESM1_ESM.pdf]

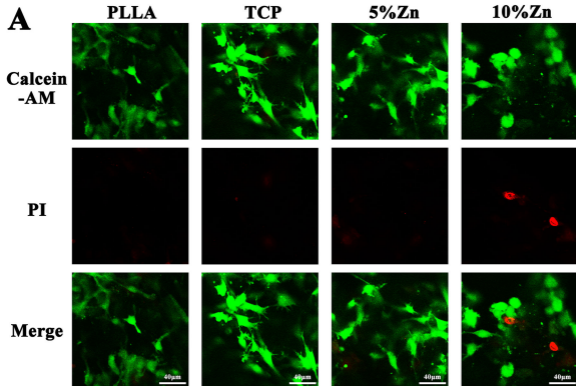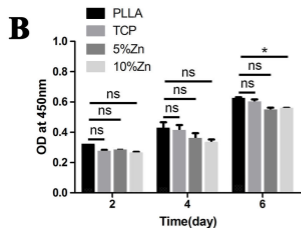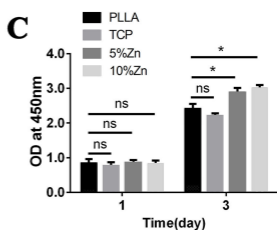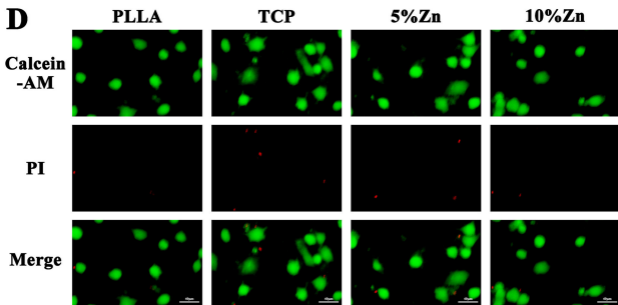

Supplement: Supplementary file 3 — Additional file 3: Figure S3. Influence of scaffolds on cytotoxicity and proliferation of incorporated PDPCs and M$\varphi$φs. Live/dead staining of PDPCs (A) and M$\varphi$φs (D) incorporated in scaffolds on days 6 and 3, respectively. Living cells were stained with calcein AM (green fluorescence), and dead cells were stained with PI (red fluorescence). Scale bar = 40 μm. Proliferation of PDPCs (B) and M$\varphi$φs (C) in scaffolds as determined by CCK-8 assays during 6-day and 3-day incubation periods, respectively. PDPCs, periosteum-derived progenitor cells; M$\varphi$φ macrophages, ns no significance; *p < 0.05; **p < 0.01. [file 12951_2021_956_MOESM3_ESM.pdf]

**4 weeks**

**8 weeks**

**PLLA&TCP**

**5%Zn&10%Zn**

**PLLA&TCP**

**5%Zn&10%Zn**

**Heart**

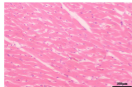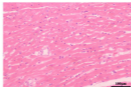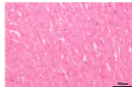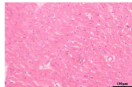

**Liver**

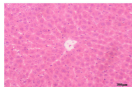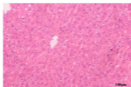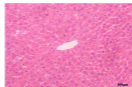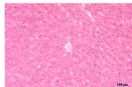

**Spleen**

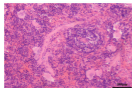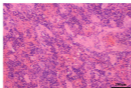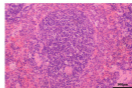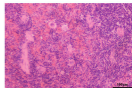

**Lung**

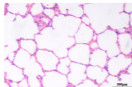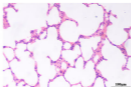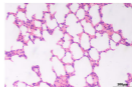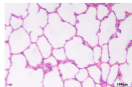

**Kidney**

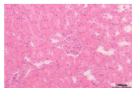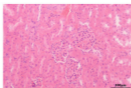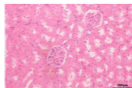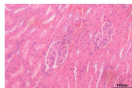

Supplement: Supplementary file 4 — Additional file 4: Figure S4. Toxicity evaluation of scaffolds in vivo. Tissue sections of the heart, liver, spleen, lung and kidney from rats treated with scaffolds were analyzed via H&E staining. Scale bar = 100 μm. [file 12951_2021_956_MOESM4_ESM.pdf]
