# Supplementary material for: Sustained zinc release in cooperation with CaP scaffold promoted bone regeneration via directing stem cell fate and triggering a pro-healing immune stimuli
Source: J Nanobiotechnology. 2021 Jul 12;19:207. doi: 10.1186/s12951-021-00956-8 (PMC8274038; doi:10.1186/s12951-021-00956-8)
Supplement: Supplementary file 2 — Additional file 2: Figure S2. Schematic illustration of PDPC-seeded A and M$\varphi$φ-seeded B scaffolds (PLLA, TCP/PLLA, 5%Zn/TCP/PLLA, and 10%Zn/TCP/PLLA) [file 12951_2021_956_MOESM2_ESM.pdf]

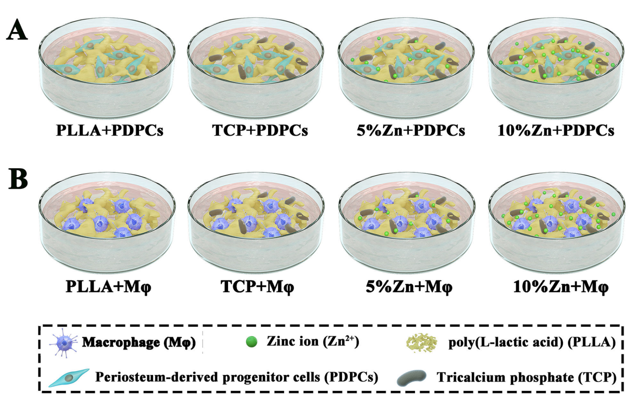
A
PLLA+PDPCs
TCP+PDPCs
5%Zn+PDPCs
10%Zn+PDPCs
B
PLLA+Mφ
TCP+Mφ
5%Zn+Mφ
10%Zn+Mφ
Macrophage (Mφ)
Zinc ion (Zn2+)
poly(L-lactic acid) (PLLA)
Periosteum-derived progenitor cells (PDPCs)
Tricalcium phosphate (TCP)
